# Education and information needs for physicians about rare diseases in Spain

**DOI:** 10.1186/s13023-019-1285-0

**Published:** 2020-01-17

**Authors:** Enrique Ramalle-Gómara, Elena Domínguez-Garrido, María Gómez-Eguílaz, María Eugenia Marzo-Sola, José Luis Ramón-Trapero, Josefa Gil-de-Gómez

**Affiliations:** 1 Department of Epidemiology, La Rioja Government, La Rioja, Spain; 2 Rioja Health Foundation, La Rioja, Spain; 3 Neurology Department, San Pedro Hospital, La Rioja, Spain; 4 Primary Care Service, La Rioja, Spain; 5 Teaching Department of Specialized Health Training, La Rioja, Spain

**Keywords:** Rare diseases, Education and information needs, Clinicians, Survey

## Abstract

**Background:**

Rare diseases are a priority objective for public health systems. Given its complexity, late and misdiagnoses occur very often which causes mental and physical burden for patients and family. This would be caused, in part, for unprepared clinicians in this field. The aim of this study was to report the training needs and the perceived shortcomings of Spanish physicians of the public health system in the diagnosis, treatment and monitoring of patients with rare diseases.

**Methods:**

We used a descriptive cross-sectional study through an “ad hoc” survey of 26 questions was completed by 132 primary care physicians and 37 specialists during April and May 2018.

**Results:**

Less than a third of the physicians had received training in rare disease during their undergraduate or postgraduate years, and for hospital professionals, they received more training in the postgraduate period.

**Conclusion:**

Primary care physicians and specialists showed low training level in rare diseases. An academical and continuous program on rare disease, as well as, multidisciplinary units and high quality practice guidelines are necessary to help to prevention and support clinical decisions and improve quality of care of patients and families.

## Background

According to the European Union (EU), a disease is considered rare when it affects no more than one person in 2000. Rare diseases (RDs) are serious, chronic, and often life-threatening conditions [1]. It has been estimated that from 6 to 8% of the population will be affected by a rare disease [2]. This means that in EU between 27 to 36 million individuals will be affected by these diseases [3]. In the UK, it has been estimated that 1 in 17 individuals may have a rare disease throughout their lives [4]. A pilot study in Spain, covering 80% of the population, detected a total of 824,399 rare disease cases [5].

These diseases are an important challenge that affects public health, the development of new diagnostic methods and therapies, and the clinical, social and health care that these patients require. A recurrent concern of these patients and their families is the limited knowledge that physicians have about it due to the high clinical complexity, which results in late diagnosis and misdiagnosis [6]. The average time between the onset of symptoms and the diagnosis of a patient with a rare disease has been estimated to be close to 6 years, while in the pediatric age it is longer than 15 months [7]. This situation can be frustrating for both health professionals and patients.

Huete et al. [8] showed that the lack of specific training of health professionals in this field means that 78.8% of those affected patients have not received appropriate care, while 56% did not receive correct care. According to Kopeć et al. [9], healthcare workers reported that physicians and medical students have insufficient knowledge and limited training in rare diseases; even parents report that they often educate their pediatrician/physician about their child’s rare disease [10].

Professionals need specialized training focused on the acquisition and maintenance of the necessary competencies for an adequate care of these patients and families, and improved communication processes with an assertive, informed, involved and interactive patient in the treatment process [11].

In 2009, the Council of the European Union [12] attempted to improve training in rare diseases and recommended “…*sufficient education and training for all health professionals, to make them aware of the existence of these diseases and resources that are available to them* “, as well as “ …*the development of medical training in areas related to the diagnosis and management of rare diseases, such as genetics, immunology, neurology, oncology or pediatrics* “. Likewise, in Spain, in its 2014 update, the Strategy on Rare Diseases of the Spanish National Health System [13] recognizes that it is necessary to improve the training of health professionals, beginning with the identification of their training needs. No information about these needs is available for healthcare professionals in Spain. In a previous study, we found differences in rare diseases knowledge from resident doctors to health and non-health future professionals [14]. Avellaneda et al. [15] described that only 20% of physicians had carried out specific training and only 15% have a good knowledge about rare diseases.

Our objective is to report the training needs and the perceived shortcomings of Spanish physicians of the public health system in the prevention, diagnosis, treatment and monitoring of patients with rare diseases.

## Methods

Descriptive cross-sectional study through self-reported surveys (Additional file 1) by La Rioja (Spain) public health system medical doctors from primary care and specialized care services (oncology, hematology, neurology, pediatrics and obstetrics and gynecology). These were selected as they have the highest rate of rare disease patients.

The sample size was calculated based on a report from Esteban et al., where they provide an average of 2.12 (SD: 0.86) values in a Likert scale about information needs/specific training in rare diseases. Then, from a total of 261 primary care physicians, a sample size of 177 individuals would be necessary to achieve an accuracy of 0.07 units in the estimation of a mean with a bilateral confidence interval of 95% with a finite population correction.

On the other hand, a total of 87 specialized physicians (10 hematologists, 33 obstetricians and gynecologists, 10 oncologists, 13 neurologists and 21 pediatricians) were invited to participate in the study.

A questionnaire was designed based on two previous publications [16, 17]. We selected the questions about practice setting, clinical experience with rare disease patients, difficulties encountered when looking after patients with rare diseases, education received about rare diseases and current use of information resources in clinical practice from Zurynski et al. [16], and individual assessment of training in rare diseases from Esteban et al. [17]. In total, 13 questions in Likert scale format with answers from 1 to 5 were used. Five questions evaluate the training and information received about rare diseases, next 5 questions about knowledge of the centres and units in Spain dedicated to this field and the last 3 questions about the evaluation of social resources and patients’ associations knowledge. Highest possible score was 65 where a higher score indicates better knowledge about these questions.

Finally, we designed three specific questions about the kind of training on rare diseases respondents would like to receive. The questions were: 1) Would you like to receive training on inheritance or genetic counselling? 2) Would you like to receive training in diagnosis and treatment? 3) Would you like to receive information about websites or sources of information? The answer was a Likert scale of 1 to 5.

The questionnaire was delivered by email and their responses were manual and anonymous.

### Statistical analysis

Means and standard deviations were calculated for quantitative variables and frequencies for qualitative findings. Means were compared by t-Student test or ANOVA. Scheffé’s post-hoc test was used to adjust for multiple comparisons. The differences between proportions were assessed by chi-square test for contingency tables. Fisher’s test was used when appropriate.

In order to assess the independent association of each covariate on the total score of the scale, a multiple linear regression analysis was carried out. The regression coefficients show the effect of each category of the independent variable on the dependent variable (total score) in relation to the reference category, adjusted for the rest of the variables included in the model. All analyses were conducted using R Commander. Two-tailed test were used and *p*-values < 0.05 were considered to be statistically significant.

## Results

From 264 sending surveys, we received a complete answered questionnaire of 169 (64,0%) physicians, 132 (78.1%) from primary care and 37 (21.9%) from a hospital setting. Ninety-nine women (58.6%) and 70 men (39.9%), with a mean age of 50.7 years (SD, 9.0). The mean of professional practice was 24.4 years (SD, 9.0). By medical specialty, primary care was divided by area in 59 (34.9%) urban and 73 (43.2%) rural, and from hospital department, 5 (3.0%) were from gynecology, 6 (3.6%) hematology, 10 (5.9%) neurology, 9 (5.3%) oncology, and 7 (4.1%) pediatrics.

Less than a third of the physicians had received training in rare diseases during their undergraduate or postgraduate years, and for hospital professionals, they received more training in the postgraduate period. Less than 40% had attended continuing education courses in the last 5 years. Sixty-five (36.4%) did a specific course on rare diseases in the last 5 years and 61 (83.6%) considered the courses useful (Table 1).

**Table 1:** Characteristics of physicians and their practice (Based in Zurynski et al.2017)

Variable	Hospital	Primary care	*p*-value
	(*n* = 37)	(*n* = 132)	
Age (Mean, SD)	46.1 (8.5)	52.0 (8,8)	**0.001**
Years of practice (Mean, SD)	19.9 (7.9)	25.6 (9.0)	**0.001**
Rare diseases included in medical degree (N, %)	10 (27.0)	35 (26.5)	0.832
Rare diseases included in post-graduate medical education (N, %)	19 (51.4)	25 (18.9)	**0.001**
Continuing Medical Education in the last 5 years about rare diseases (N, %)	17 (45.9)	54 (40.9)	0.765
Number of rare disease patients seen during clinical career (N, %):			
0	0 (0.0)	7 (5.3)	**0.002**
1--4	6 (16.2)	59 (44.7)	
5--10	8 (21.6)	26 (19.7)	
11--15	5 (13.5)	8 (6.0)	
16--20	2 (5.4)	6 (4.5)	
> 20	10 (27.0)	20 (15.2)	
> 100	6 (16.2)	5 (3.8)	

*Significant difference *p* < 0.05

The main difficulties in the care of patients with rare diseases are derived from the lack of diagnostic guidelines, delay or inability to make a definitive diagnosis and uncertainty about where to refer the patient to. One significant difficulty was the lack of available treatments (78.4% vs. 36.4%; *p*-value = 0.001) and difficulty accessing new drugs or therapies currently available overseas, not yet licensed in Spain (45.9% vs 15.2%, *p*-value = 0.001) (Table 2).

**Table 2:** Difficulties encountered by physicians while caring for patients with rare diseases (Based in Zurynski et al.2017)

Variable (N, %)	Hospital	Primary care	*p*-value
	(*n* = 37)^a^	(*n* = 132)^a^	
Lack of diagnostic guidelines	26 (70.3)	78 (59.1)	0.211
Lack of access to diagnostic tests	12 (32.4)	44 (33.3)	0.918
Delay or inability to make a definitive diagnosis	23 (62.2)	89 (67.4)	0.552
Lack of treatment or management guidelines	21 (56.8)	77 (58.3)	0.864
Lack of available treatments	29 (78.4)	48 (36.4)	**0.001**
Difficulty accessing new drugs or therapies currently available overseas, not yet licensed in Spain	17 (45.9)	20 (15.2)	**0.001**
Uncertainty about where to refer patients to	23 (62.2)	88 (66.7)	0.612
Difficulties in accessing allied health services (Physiotherapy, psychology,...)	9 (24.3)	41 (31.1)	0.421
Difficulties in accessing genetic testing	12 (32.4)	42 (31.8)	0.944
Uncertainty about available peer support groups for the patient and family	14 (37.8)	59 (44.4)	0.476

^a^Significant difference *p* < 0.05

When we evaluated questions about knowledge and management of rare diseases we found that the overall score does not reach a third of the maximum score, 28.16 for hospital professionals and 22.19 for primary care (Table 3).

**Table 3:** Likert questions about training for the management and knowledge of rare diseases.(Based in Esteban Bueno et al. 2015)

Questions	Hospital (*n* = 37) Mean (SD)	Primary care (*n* = 132) Mean (SD)	*p*-value
I consider that the medical training (clinical aspects) that I have received on rare diseases is adequate	2.29 (0.94)	1.72 (0.96)	**0.001**
I believe that the training I have received on the psychosocial impact of rare diseases is adequate	1.77 (0,81)	1.71 (0.93)	0.730
I consider myself qualified to coordinate the health care of a patient with a rare disease	2.40 (1.17)	1.82 (1.02)	**0.002**
I know the protocol of action that I must follow with a patient with a rare disease	2.19 (1.12)	1.58 (0.83)	**0.002**
I feel qualified to give genetic counselling to my patients with rare diseases	2.02 (1.01)	1.41 (0,74)	**0.001**
I know the existence of some of the registries of rare diseases existing in our country	2.33 (1.29)	2.10 (1.26)	0.305
I have enough information about the operation of rare disease registries	1.79 (0.99)	1.58 (0.84)	0.154
I know what the Reference Units for rare diseases are	2.40 (1.22)	1.97 (1.11)	**0.029**
I know the role of these Reference Units in the monitoring of these diseases	2.38 (1.29)	1.87 (1.07)	**0.009**
I know the existence of the State Reference Center for People with rare diseases and their families	2.09 (1.23)	1.87 (1.14)	0.272
I know how to refer patients to Reference Units	2.47 (1.32)	1.53 (0.77)	**0.001**
I know the functions performed by the State Reference Center for People with Rare Diseases and their Families	1.95 (1.07)	1.53 (0.78)	**0.019**
I know the global / national organizations / associations working in the field of rare disease	2.26 (1.09)	1.58 (0.85)	**0.001**
Overall	28.16 (10.55)	22.19 (8.77)	**0.001**

*Significant difference *p* < 0.05

**Fig. 1:**
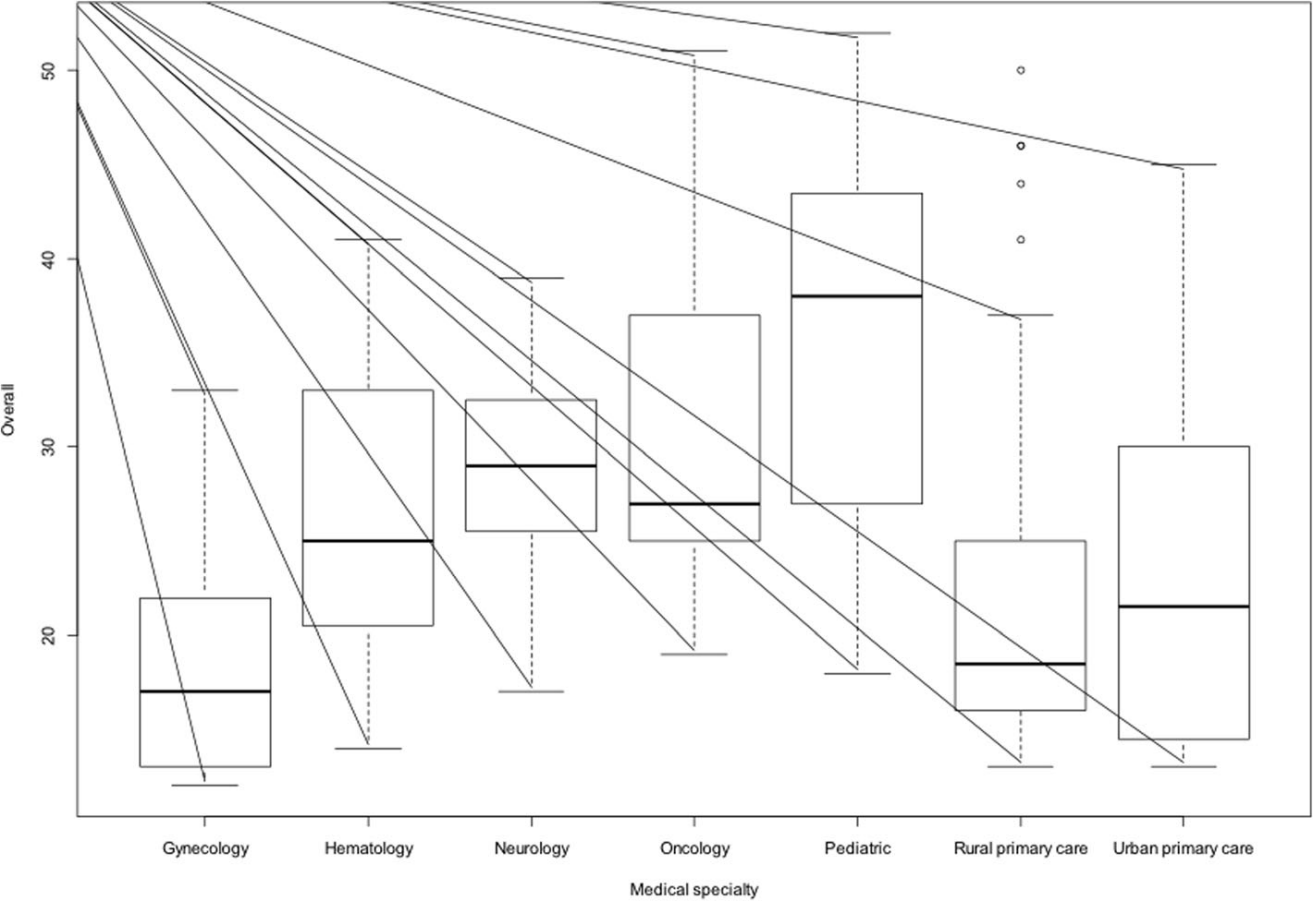
Overall score and medical specialty

Regarding medical speciality, primary care and gynecologists have lower scores than their colleagues, and pediatricians showed better abilities to care for rare diseases. (Table 4 and Fig. 1).

**Table 4:** Overall score and medical specialty

Medical specialty	Mean	SD
Gynecology	18.7	7.5
Hematology	26.7	9.4
Neurology	28.5	6.9
Oncology	32.6	10.9
Pediatric	35.6	12.1
Rural primary care	21.6	8.7
Urban primary care	22.9	8.8

*Differences (*p* < 0.05) between:

Oncology and pediatric vs urban primary care

Oncology and pediatric vs rural primary care

Oncology vs gynecologic

Pediatric vs gynecologic

Multiple linear regression model on the total score of the questionnaire indicated that specialists that received training courses improved the overall score (Table 5).

**Table 5:** Multiple linear regression: factors related to overall score

Variable	Coefficient	*p*-value
Men vs women	−0.293	0.817
Years of practice	−0.009	0.897
Hospital vs primary care	5178	**0.001**
Continuing vs No Continuing medical education	6738	**0.001**

*Significant difference *p* < 0.05

Finally, all physicians showed a strong interest to receive training, especially in genetic counselling, 4.1 and 4.0 out of 5 points for primary care and hospital physicians respectively (*p* = 0.75). Regarding diagnosis and treatment, both groups scored 4.2 and with regards to the need to receive information on rare diseases websites, score was 4.4 for primary and 4.2 for specialization (*p* = 0.19).

## Discussion

To develop effective continuing medical education (CME) strategies that can impact clinical practice, information should initially be gathered to assess the needs and attitudes of the target group, followed by an evaluation of these educational strategies on practice [18]. Our study has shown a low degree of training of physicians, both for primary and specialized care, in the care of patients with rare diseases. The paradox of rare diseases, although individually rare, collectively affects a significant proportion of the general population, thus reflecting the well-known concept that “little drops of water will make the mighty ocean” [19]. In Australia, approximately 8% of the population live with any one of about 10,000 known rare diseases. This is similar to the proportion of people living with diabetes or asthma [20].

Therefore, most physicians will face the diagnosis or treatment of a rare disease at some point in their professional lives. In our study, almost 90% of the clinicians had cared for these patients in their professional career. In the USA it has been estimated that 1.6% of outpatient visits by primary care physicians are to care for these patients [21].

The lack of training is also perceived by patients or their families, who become experts in their disease [11]. This is a challenge for their clinicians who are unaccustomed to their patients knowing more about an illness than they do. Listening to patients / parents always with belief in what they are saying is the key to both unlocking red flags quickly and giving patients confidence in their GP. When patients were asked what was the one thing their GP could have done to improve the diagnostic journey, “Believing me” is the number one response [22]. The existence of clinical practice guidelines can improve clinical decision making and public health [23]. Few years ago, some specialized webs offer this content in Spanish, as an example, *Enfermedades Raras-Orphanet* (https://www.orpha.net).

Doctors are not aware of socio-health resources or referral centres available for these patients. The scores on the scale do not reach 3 points, as Esteban et al. pointed out [17]. Therefore, the difficulties in making an adequate diagnosis, prescribing treatment or guiding patients about social and health care or about where the referral centres may be indicated that there are still difficulties for an adequate management of RD, as described above. Avellaneda et al. [15] show that primary health care physicians have a low level of knowledge of RD, although a high interest, with emphasis on primary prevention, the importance of the family environment, genetic counselling and health education. Challenges for clinicians who care for affected individuals include gaining knowledge and experience in caring for such patients [24]. And important issue is being the contribution of the European Reference Networks (ERN), that bring all existing knowledge, experience and resources together, using three e-health and telemedicine tools [25, 26].

The lack of diagnostic and treatment guidelines and the difficulty in accessing genetic tests limit the possibilities of making an accurate and rapid diagnosis. Schieppati et al. [27] found that in 25% of the cases the diagnostic delay was from 5 to 30 years. In Spain the Spanish Undiagnosed Rare Diseases Program (SpainUDP) has been launched. SpainUDP offers a multidisciplinary approach to those patients who have long sought a diagnosis without any success [28].

About 46% of hospital doctors expressed that they have difficulty accessing treatments. Zurynsky’s study [16] also found that about 40% of pediatricians had difficulty accessing treatments. It is unrealistic to consider that a doctor can know all the rare diseases described at this moment, but patients described the “diagnostic odyssey” to refer to that long process, often decades, that takes them from hospital to hospital, with misdiagnoses and inappropriate treatments [29]. Other countries have some experience with specific training in rare diseases. In France, all health professionals, medical doctors, midwives, nurses and paramedics attend a 2-h training session on rare diseases, which raises awareness and identifies sources of information on rare diseases for health professionals [20]. In Spain, it has been claimed for a long time that rare diseases are included in undergraduate Medicine and Nursing curricula and in postgraduate and continuing medical training [2].

The need to receive training on genetic counselling, diagnosis and treatment of RD, as well as information on web pages is clear, with scores very close to the maximum. A recent study by Vandeborne et al. [30] showed that when participants were asked if they needed rare disease information, 83% of the GPs, 95% of pediatricians, 97% of adult specialists and 100% of pediatric specialists indicated a need to have information on rare diseases.

There are some differences between primary care and hospital doctors. In general, the perceived training and knowledge of the resources available for the care or referral of patients is greater for hospital doctors. The reason, probably, is that the number of rare diseases faced by a hematologist or pediatrician is much smaller than those potentially seen by a General Practitioner. In a large survey directed at 837 patients with rare diseases, parents, and spouses and 531 health care professionals, it was also found that those respondents considered primary care physicians were much more likely to rate their level of knowledge as fair or poor (56.4%) compared with respondents who were considered specialists (6.0%) [10]. When physicians were specifically asked about their training, most (56.7%) of the primary care respondents rated their training as neutral, ineffective, or very ineffective, compared to 40% of specialists [10]. Also, in Bulgaria, primary care physicians have a low level of general knowledge and awareness [31].

The number of physicians who had received continuing training in RD is less than 40%, although this figure is double of that reported by Avellaneda et al. [15]. Although this percentage is not very high, the study has also shown that doctors who had received continuing education courses felt better prepared and more knowledgeable to care for the sick. This result shows the need to implement ongoing training programs to improve the degree of clinical knowledge and diagnosis related to RD, as well as to publicize the existence of socio-health-type resources and patient associations among physicians. In 2006, primary care physicians in Spain created a working group on rare diseases to improve care for these patients [32].

There are some limitations to this study. Firstly, our hospital is a medium-sized centre (630 beds) and it is not a reference hospital for the diagnosis or treatment of rare diseases. Second, the response rate was 64.0%. No significant differences between respondents and non-responders were found in clinicians age, years of professional practice, sex, or work environment. We assume, therefore, that the non-response is random and not introduce a qualitative bias in the results.

## Conclusion

In conclusion, the study supports other investigations that have shown that clinicians lack easy access to educational opportunities and information resources regarding rare diseases. It is imperative that the public health system includes ongoing training on rare diseases in programs to improve the training of physicians in both primary care and specialized care.

## Supplementary information

**Additional file 1.** Questionnaire

## Data Availability

See additional files.

## Abbreviations

CME: Continuing medical education
ERN: European Reference Network
EU: European Union
FEDER: Fondo Europeo de Desarrollo Regional
GP: General Practitioner
RD: Rare disease
SD: Standar Desviation
Spain UDP: Spanish Undiagnosed Rare Diseases Program
UK: United Kingdom
USA: United States of America
