# Supplementary material for: Education and information needs for physicians about rare diseases in Spain
Source: Orphanet J Rare Dis. 2020 Jan 17;15:18. doi: 10.1186/s13023-019-1285-0 (PMC6969468; doi:10.1186/s13023-019-1285-0)
Supplement: Supplementary file 1 — Additional file 1. Questionnaire [file 13023_2019_1285_MOESM1_ESM.pdf]

Anexo 1. Encuesta

1. Sexo: ☐ 0. Mujer
☐ 1. Varón

2. Edad:

3. Años de ejercicio profesional:

4. Ámbito laboral: ☐ 0. Hospital *(Pasar a pregunta 6)*
☐ 1. Atención Primaria

5. Centro de salud en Logroño: ☐ 0. No
☐ 1. Sí

A continuación, marque con una x la respuesta que considere

6. ¿Se incluyeron las enfermedades raras en su plan de estudios de la Licenciatura o Grado en Medicina?

☐ 0. No
☐ 1. Sí
☐ 2. No lo recuerdo

7. ¿Durante el periodo de formación MIR se formó sobre enfermedades raras?

☐ 0. No
☐ 1. Sí
☐ 2. No lo recuerdo
☐ 3. No hice el MIR

8. ¿Ha asistido a conferencias, cursos, congresos, jornadas, talleres educativos, etc. sobre enfermedades raras en los últimos 5 años?

☐ 0. No (Pasar a pregunta 10)
☐ 1. Sí
☐ 2. No lo recuerdo (Pasar a pregunta 10)

9. ¿Fueron estas conferencias, talleres, etc., útiles para mejorar sus competencias sobre las enfermedades raras?

☐ 0. No
☐ 1. Sí
☐ 2. No lo recuerdo

10. ¿Alguna vez ha atendido a un paciente con un diagnóstico de enfermedad rara?

☐ 0. No

☐ 1. Sí
☐ 2. No estoy seguro

11. Durante el curso de su práctica clínica calcule aproximadamente cuántos de sus pacientes han tenido una enfermedad rara

☐ 0. Ninguno
☐ 1. De 1 a 4 pacientes
☐ 2. De 5 a 10 pacientes
☐ 3. De 11 a 15 pacientes
☐ 4. De 15 a 20 pacientes
☐ 5. Más de 20 pacientes
☐ 6. Más de 100 pacientes

12. ¿Alguna vez ha atendido a un paciente con un grupo inusual de signos y síntomas para los cuales fue difícil establecer un diagnóstico definitivo?

☐ 0. No
☐ 1. Sí
☐ 2. No estoy seguro

13. ¿Cuándo fue la última vez que atendió a un paciente nuevo con cualquier enfermedad rara diagnosticada o con un grupo de signos o síntomas no diagnosticados?

☐ 0. Menos de 6 meses
☐ 1. De 6 a 12 meses
☐ 2. De 1 a 3 años
☐ 3. Más de 3 años
☐ 4. No he atendido a ninguno (Saltar a pregunta 24)

Si alguna vez ha diagnosticado o tratado a pacientes con enfermedades raras, ¿en cuál de las siguientes áreas encontró dificultades? (**Marque con una X. Puede seleccionar más de una**)

14 Falta de guías clínicas de diagnóstico	
15. Falta de acceso a pruebas diagnósticas	
16. Retraso o incapacidad para hacer un diagnóstico definitivo	
17. Falta de guías clínicas de tratamiento o de pautas de manejo	
18. Falta de tratamientos disponibles	
19. Dificultad para acceder a nuevos medicamentos o terapias que se usan actualmente en el extranjero, que aún no tienen licencia en España	
20. Incertidumbre sobre a qué personas o centros de referencia consultar	
21. Dificultad para acceder a servicios de salud complementarios (por ejemplo fisioterapia, logopedia, psicología, etc.)	
22. Dificultad para acceder a pruebas genéticas	
23. Incertidumbre sobre los grupos de apoyo y asociaciones de enfermos disponibles para el paciente y su familia	

Anote el grado de acuerdo con estas afirmaciones, teniendo en cuenta que 1 es completamente en desacuerdo y 5 completamente de acuerdo (**ponga en otro color o rodee con un círculo la puntuación elegida**)

24. Considero que la formación médica (aspectos clínicos) que he recibido sobre las enfermedades raras es adecuada	1	2	3	4	5
25. Considero que la formación que he recibido sobre la repercusión psicosocial de las enfermedades raras es adecuada	1	2	3	4	5
26. Me considero capacitado para coordinar la atención sanitaria de un paciente con una enfermedad rara	1	2	3	4	5
27. Conozco el protocolo de actuación que debo seguir ante un paciente con una enfermedad rara	1	2	3	4	5
28. Me siento capacitado para dar asesoramiento genético a mis pacientes con enfermedades raras	1	2	3	4	5
29. Conozco la existencia de alguno de los registros de enfermedades raras existentes en nuestro país	1	2	3	4	5
30. Tengo información suficiente sobre el funcionamiento de los registros de enfermedades raras	1	2	3	4	5
31. Sé lo que son las Unidades de Referencia para las enfermedades raras	1	2	3	4	5
32. Conozco el papel que desempeñan estas Unidades de Referencia en el seguimiento de estas enfermedades	1	2	3	4	5
33. Conozco la existencia del Centro de Referencia Estatal de Atención a Personas con enfermedades raras y sus familias	1	2	3	4	5
34. Conozco la forma de derivar a los pacientes a las Unidades de Referencia	1	2	3	4	5
35. Conozco las funciones que desempeña el Centro de Referencia Estatal de Atención a Personas con enfermedades raras y sus Familias	1	2	3	4	5
36. Conozco las organizaciones/asociaciones mundiales/nacionales que trabajan en el ámbito de las enfermedades raras	1	2	3	4	5
37. ¿Le gustaría recibir formación sobre herencia o asesoramiento genético en relación a las enfermedades raras?	1	2	3	4	5
38. ¿Le gustaría recibir formación sobre diagnóstico y tratamiento de las enfermedades raras?	1	2	3	4	5
39. ¿Le gustaría recibir información sobre páginas webs o fuentes de información sobre enfermedades raras?	1	2	3	4	5
